# Detection and characterization of Deformed Wing Virus (DWV) in apiaries with stationary and migratory management in the province of Entre Ríos, Argentina

**DOI:** 10.1038/s41598-024-67264-7

**Published:** 2024-07-20

**Authors:** F. N. Gonzalez, F. Raticelli, C. Ferrufino, G. Fagúndez, G. Rodriguez, S. Miño, M. J. Dus Santos

**Affiliations:** 1 Instituto de Virología e Innovaciones Tecnológicas (IVIT), CICVyA, Instituto Nacional de Tecnología Agropecuaria. De las Cabañas y De los Reseros s/n, Hurlingham, Buenos Aires, Argentina; 2 Laboratorio de Especialidades Productivas de Maciá (LEPMA), Ecología y Medio Ambiente, Secretaría de Producción, Municipio de Gobernador Maciá, Entre Ríos Argentina; 3 Laboratorio de Actuopalinología, CICYTTP (CONICET - UADER), Diamante, Entre Ríos Argentina; 4 EEA Hilario Ascasubi, Instituto Nacional de Tecnología Agropecuaria, Ruta Nacional 3, Km 794, Hilario Ascasubi, Buenos Aires Argentina; 5 EEA Cerro Azul, Instituto Nacional de Tecnología Agropecuaria, Ruta Nacional 14, Km 836, Cerro Azul, Misiones Argentina

**Keywords:** Honey bee, Pathogen, Deformed wings virus, Stationary and migratory beekeeping, *Apis mellifera*, Genomic analysis, Virology

## Abstract

In Argentina, migratory activity in search of floral diversity has become a common approach to maximizing honey production. The Entre Ríos province possesses a floral diversity that allows beekeepers to perform migratory or stationary management. Beyond the impact caused by transhumance, migratory colonies in this province start and end the season in monoculture areas. To study the effect of these practices on viral infection, we assayed for the presence, abundance and genetic characterization of the Deformed Wing Virus (DWV) in honey bees from apiaries with both types of management. In migratory apiaries, DWV was detectable in 86.2% of the colonies at the beginning of the season (September 2018), and 66% at the end of the season (March 2019). On the other hand, DWV was detected in 44.11% and 53.12% of stationary samples, at the beginning and the end of the season, respectively. Sequence analysis from migratory and stationary colonies revealed that all samples belonged to DWV-A type. The highest viral loads were detected in migratory samples collected in September. Higher DWV presence and abundance were associated with migratory management and the sampling time. Based on our findings we propose that the benefit of migration to wild flowering areas can be dissipated when the bee colonies end the season with monoculture.

## Introduction

Globally, honey bees play a crucial role in pollinating agricultural crops and wild plant species, thereby contributing to landscape biodiversity enhancement and the production of honey^1,2^.

A growing body of evidence indicates that the health of commercially managed honey bee colonies is influenced by multiple biotic and abiotic factors that can act synergistically and be exacerbated by apicultural practices^3–5^. These factors include parasites, bacterial and fungal brood pathogens^6–8^, viruses^9^, the mite *V. destructor*^10^, chemicals, poor nutrition, climate, reduced genetic diversity^11^, queen failure^12^, and management practices^13–21^.

Viruses pose a significant threat to honey bee health, leading to substantial losses in honey production and contributing to high levels of morbidity and mortality among native and wild bee populations^14,22,23^. While viral infections typically remain covert, they can manifest clinical signs of disease and weaken colonies under stressful conditions, immunosuppression, or nutritional deficiencies.

The Deformed Wing Virus (DWV) is one of the most important viruses that affect honey bees due to its worldwide prevalence and association with colony collapse disorder^24^. DWV presents different transmission routes; horizontal transmission is mediated by trophallaxis, cannibalism, and cleaning, while vertical transmission can be conducted by either drones or the queen. In addition, the mite *Varroa destructor* acts as an efficient vector when it is parasitizing the honey bee. This virus infects all castes and has been detected in different stages of *A. mellifera* (egg, larvae, pupae, and adult). DWV´s overt infections are associated with the appearance of bees with deformities in their wings, behavioral symptoms, and a short life expectancy, which is manifested at the colony level by a progressive decrease in the population size^25,26^. DWV is a ssRNA virus classified within the genus *Iflavirus*. It encompasses three master variants: DWV-A, DWV-B (also recognized as Varroa destructor-1 virus), and DWV-C^27–30^.

Beekeeping is carried out through two types of management: stationary or migratory. In areas where there is a wide floral diversity, stationary management is practiced, allowing the colonies to remain in the same place throughout the whole season. Colonies that move between different crops practice migratory management which aims to alleviate floral dearth, boost honey production, and minimize the need for supplemental feeding, thereby demonstrating greater financial advantages and efficiency for honey production compared to stationary beekeeping^31,32^.

Research conducted in India and Saudi Arabia revealed that inadequate bee flora during prolonged floral dearth periods can affect honey bee health and increase colony losses in stationary beekeeping managements^31^. In addition, in Europe, beekeepers who migrate their colonies in search of superior foraging resources, experience lower winter colony losses, as documented by Oberreiter & Brodschneider and van Der Zee et al.^33,34^.

The movement to new pollination locations forces the colonies to adapt to new environmental conditions, including daily oscillations in temperature, humidity, and wind patterns. Additionally, their exposure to pathogen might increase, and infections by new pathogens may occur^35,36^. In fact, transportation and pollination services have recently been proposed to increase the infestation rate and abundance of *Nosema ceranae* and some viruses in *A. mellifera* worker bees^17–21,37^. Stress experienced during transportation also impairs immunity^17^ and increases susceptibility to disease due to pollinating large monocultures^38,39^.

In studies carried out in Western US with bee colonies involved in almond pollination, it was observed that pathogen prevalence was higher in samples from weak colonies that were obtained after almond pollination^21^. Two other controlled experiments showed that migratory colonies returned with fewer bees and higher BQCV loads than stationary colonies; DWV prevalence and loads were also higher in the migratory colonies upon their return and remained so until the end of the study^19^.

In other cases, migratory beekeeping is conducted in response to climate seasonality. In a recent report, Jara et al.^18^ studied *V. destructor, Nosema* spp. and DWV infestation and infection rates before, during and after the migratory operation in Spain. They found an increased incidence of *V. destructor* and *Nosema ceranae* and a lower DWV viral load in migratory colonies. Bees exposed to migratory management during adulthood showed increased levels of the AKI virus complex (Acute bee paralysis, Kashmir bee, and Israeli acute paralysis viruses) and decreased levels of antiviral gene expression. Moreover, the seasonal increase in DWV was higher in juvenile bees from migratory colonies than in those from stationary ones^17^.

Argentina is one of the most important honey producers and exporters worldwide. In this country, migratory beekeeping is conducted in search of specific flowering with the purpose of improving nutritional resources, boosting honey production and the possibility of harvesting different honey varieties during the season^40^. In the Entre Ríos province, colonies from the south remain in a single location the whole year. In the north, however, most beekeepers start the season with the flowering of *Citrus* spp.; in the middle of the season, they move their colonies to native forest to access a diverse range of forage sources, and they return to another monoculture crop (*Eucalyptus* spp.) at the end of the season.

In this study, we assessed the impact of stationary and migratory beekeeping practices on colony population (as a proxy for colony health) and DWV point prevalence and abundance in the apiaries of Entre Ríos province. Our findings indicate that within the analyzed period the prevailing variant of DWV in this area was DWV-A. Based on our health status analysis of stationary and migratory colonies, we suggest that while migration to wildflower areas may have a positive effect on colony health, these benefits can be dissipated when the colonies end the season with monoculture crops.

## Results

### Honey bee colony monitoring

Commercially managed colonies from Entre Ríos province were monitored at the beginning and the end of the season (i.e., September 2018 to March 2019). Throughout our study, beekeepers who conducted stationary or migratory management practices indicated that they had experienneither colony losses nor a decrease in honey production.

**Figure 1:**
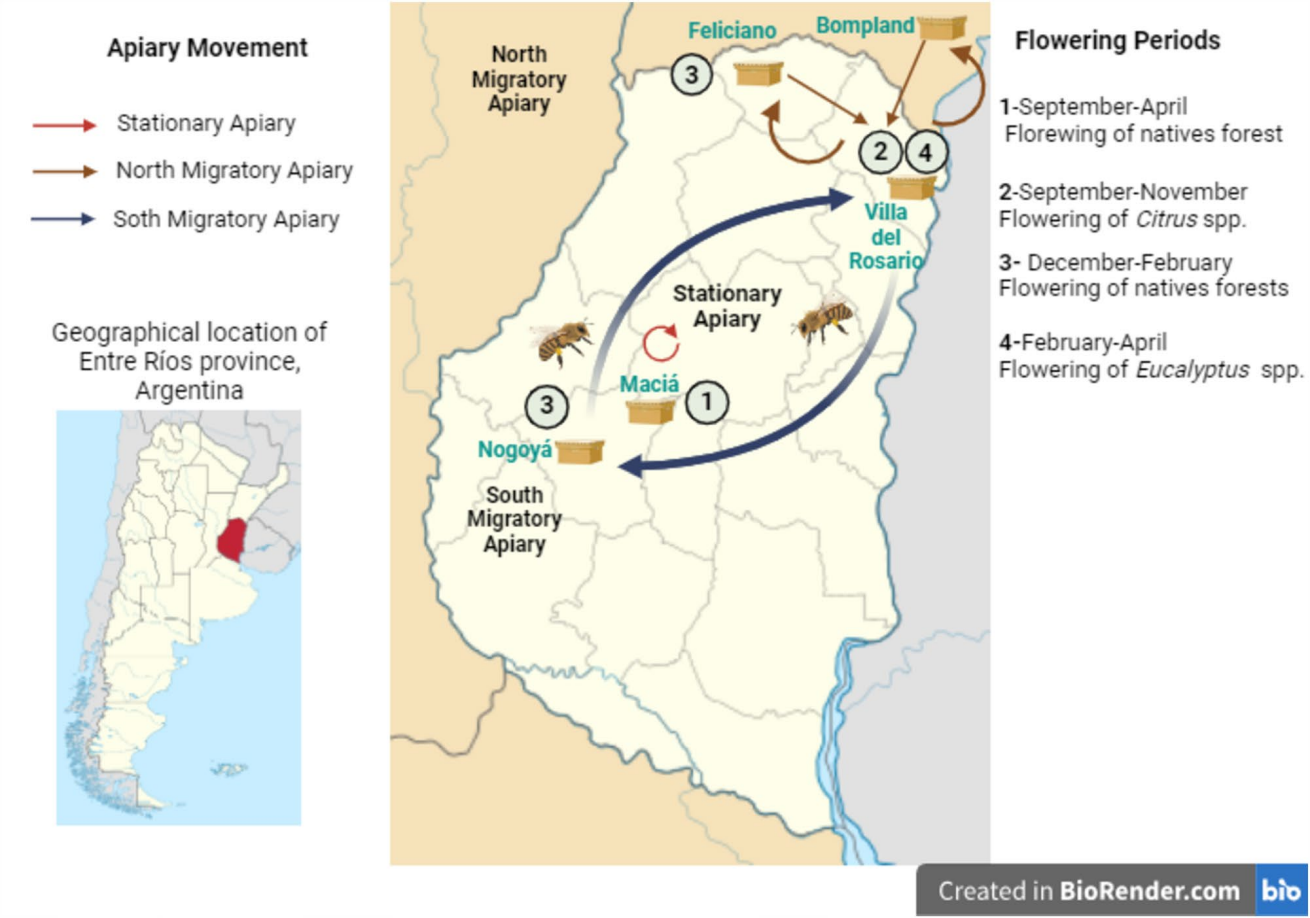
Scheme of the movement of hives and their geographical location. This diagram represents the movements undertaken by beekeepers engaged in migratory activities, as well as the fixed positions of stationary apiaries throughout the season. For migratory hives, the season begins with the flowering of *Citrus* spp. (2), which lasts for 2 months. After this flowering period, the hives migrate either northward (106–189 km, indicated by brown arrows) or southward (320 km, indicated by blue arrows) in search of more diverse floral resources, particularly native forests (3). At the end of the season, they return to their original location (Villa del Rosario) to take advantage of *Eucalyptus* spp. flowering (4). The mentioned distances correspond to the lengths of the transportation routes. In contrast, stationary hives remain in the same central zone of the Entre Ríos province throughout the entire season (1), making use of the floral diversity available in that area (highlighted by the red arrow). The map was created with BioRender. http://app.biorender.com.

Stationary apiaries were situated in Gobernador Maciá village, where 6 apiaries were sampled. On the other hand, 9 migratory apiaries located in Villa del Rosario village were selected. These migratory colonies were placed in an area with monoculture crops at the beginning of the study (flowering period 2: *Citrus* spp.). Colonies remained in Villa del Rosario from September to November, throughout the flowering season of *Citrus* spp. trees. Subsequently, they moved in search of wild flowering. Four apiaries transported their colonies 106–189 km to the north (Feliciano villages in the E. Rios province and Bompland in the Corrientes province), while the other 5 apiaries were moved 320 km to the south, to Nogoya village (flowering period 3). This latter location was situated 60 km from Gobernador Maciá, where stationary management was conducted (flowering period 1). At the end of the season, in early February, all migratory colonies returned to their starting point in Villa del Rosario (flowering period 4), where wintering took place (Fig. 1).

**Figure 2:**
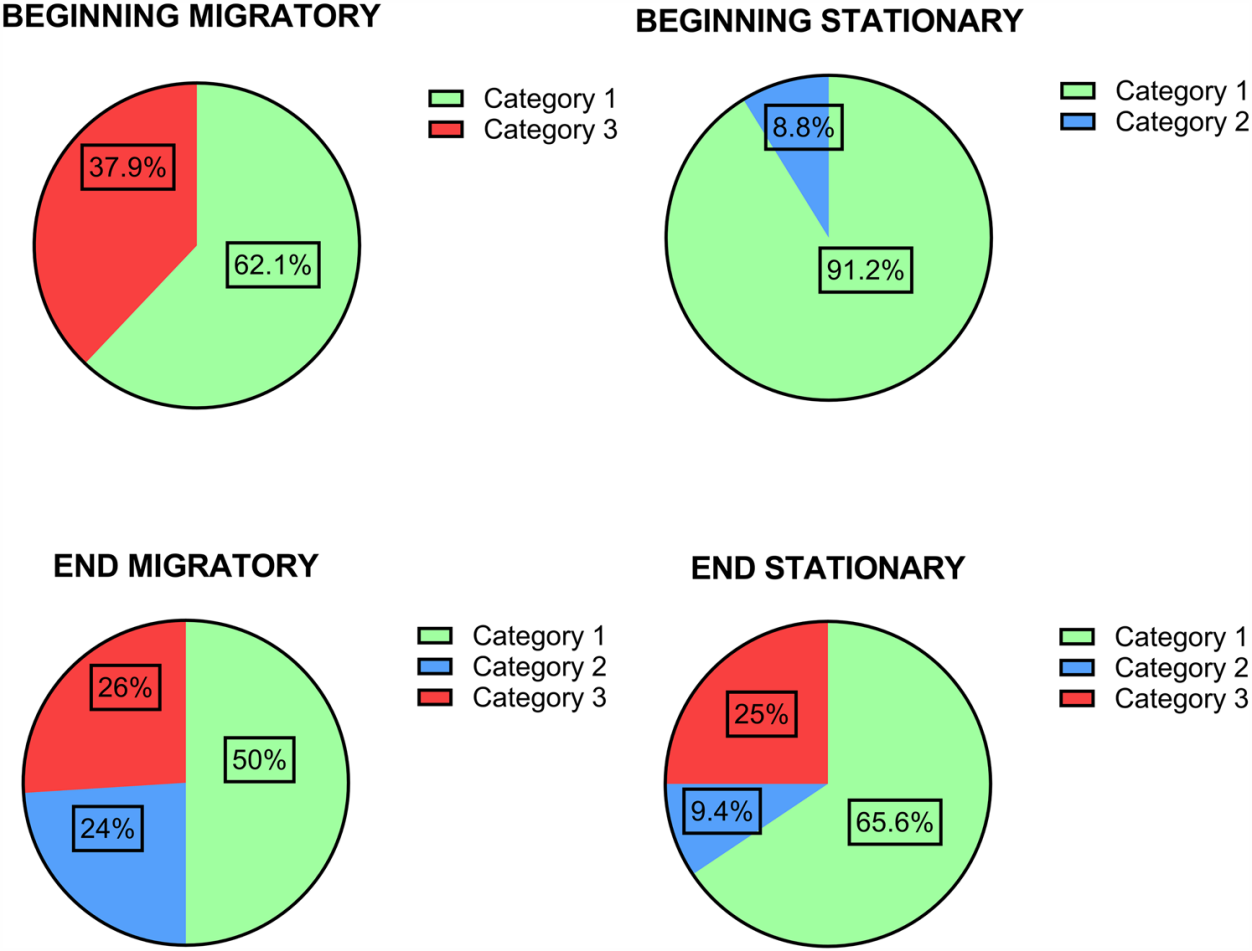
Categorization of hives in apiaries with stationary and migratory management during the 2018–2019 season. Hives were classified as strong (C1: category 1, green), medium (C2: category 2, light blue) and weak (C3: category 3, red) according to their population size, which was determined by the number of frames covered with bees. Classification at the beginning of the season: C1: 8–10 frames with bees; C2: 5–7 frames with bees; C3: < 5 frames with bees. Classification at the end of the season: C1: 7 to 8 frames; C2: 6 frames and C3: up to 5 frames. The circular graphs display the percentage of hives in each category at the beginning and the end of the season, according to the management type.

Colony population size was used as an indicator of the hive health and was monitored at each sampling event. At the beginning of the season, 34 stationary hives were sampled, with 91.2% (31/34) of the colonies classified in category C1 and 8.8% (3/34) in C2. On the other hand, out of the 32 stationary hives analyzed at the end of the season, 65.6% (21/32) belonged to category C1, 9.4% (3/32) to category C2, and 25% (8/32) to category C3. In the first sampling of migratory apiaries, 29 hives were analyzed, with 62.1% (18/29) categorized in C1 and 37.9% (11/29) in C3. At the end of the season, the hives were classified as follows: 50% (25/50) in category C1, 24% (12/50) in category C2, and 26% (13/50) in category C3 (Fig. 2 and Table 1).

**Table 1:** Description of the management conducted by migratory and sedentary apiaries during the season (September 2018-March 2019).

Sampling	Variables	Stationary apiary	Migratory apiary
Beginning	N° Hives/ N° Apiary	34/6	29/5
	Hive Category	C1: 31 hives; C2: 3 hives; C3: 0 hive	C1: 18 hives; C2: 0 hive; C3: 11 hives
	*V. destructor* control*	1	1 in each migratory movement*
	*Nosema* spp. control	No	No
	Replacement of queen	Yes	Yes
	Dietary supplement	Pollen pellets and sucrose syrup	Pollen pellets and sucrose syrup
	Type of flowering	Native forest	*Citrus* spp.
End	N° Hives/ N° Apiary	32/6	50/9
	Hive Category	C1: 21 hives; C2: 3 hives; C3: 8 hives	C1: 25 hives; C2: 12 hives; C3:13 hives
	*V. destructor* control	1	1 in each migratory movement*
	*Nosema* spp. control	No	No
	Replacement of queen	No	No
	Dietary supplement	Pollen pellets and sucrose syrup	Pollen pellets and sucrose syrup
	Type of flowering	Native forest	*Eucalyptus grandis*

*V. destructor* control*: The migratory apiaries during this work carried out 3 control treatments, one at each migratory operation.

Throughout this study, most colonies did not exhibit clinical signs associated with known pathogens. However, in 2 hives under migratory management, adult bees with deformed wings were detected in larvae and pupae at the beginning of the season.

### Varroa destructor and *Nosema* spp. diagnostics

At each sampling event, live honey bees were obtained to assess the levels of infestation with *Varroa destructor* and the presence of the microsporum parasite *Nosema* spp*.*

In all analyzed apiaries, levels of *Nosema* spp*.* were below the recommended treatment threshold (1 million spores per bee); nevertheless, stationary colonies exhibited lower levels of *Nosema* spp*.* than migratory ones, both at the beginning and the end of the season (Supplementary material 1).

In the analysis of *V. destructor*, infestation levels were also found to be below the recommended treatment application threshold (3%) in nearly all samples. Only 2 stationary and 2 migratory colonies showed infestation levels slightly above 3% at the end of the season (Supplementary material 1).

The presence of *V. destructor* in its reproductive status was also evaluated by sampling larvae and pupae whenever possible. In apiaries with both types of management, mites were found parasitizing the larvae and puppae (Table 3 and Supplementary material 2).

### DWV prevalence and abundance

In this study, DWV point prevalence was defined as the proportion of colonies that tested positive at each sampling event, while DWV abundance referred to viral RNA abundance quantified by RT-qPCR (viral load: VL). DWV was detected in all study areas and all types of samples, including adult bees, larvae, pupae, mites, and pollen. However, DWV point prevalence and abundance in honey bee colonies varied depending on the sampling date and beekeeper operations. The highest number of DWV-positive colonies (86.2%; 25/29) was obtained from honey bee samples with migratory management, collected in September when colonies were located in Villa del Rosario. The VLs in those DWV-positive colonies had a mean of 7.54 log10 genome equivalents/bee and reached a maximum value of 12.66 log10 genome equivalents/bee. Meanwhile, in stationary apiaries, 44.11% (15/34) of colonies tested positive for DWV detection and had a VL mean of 5.88 log10 genome equivalents/bee.

**Figure 3:**
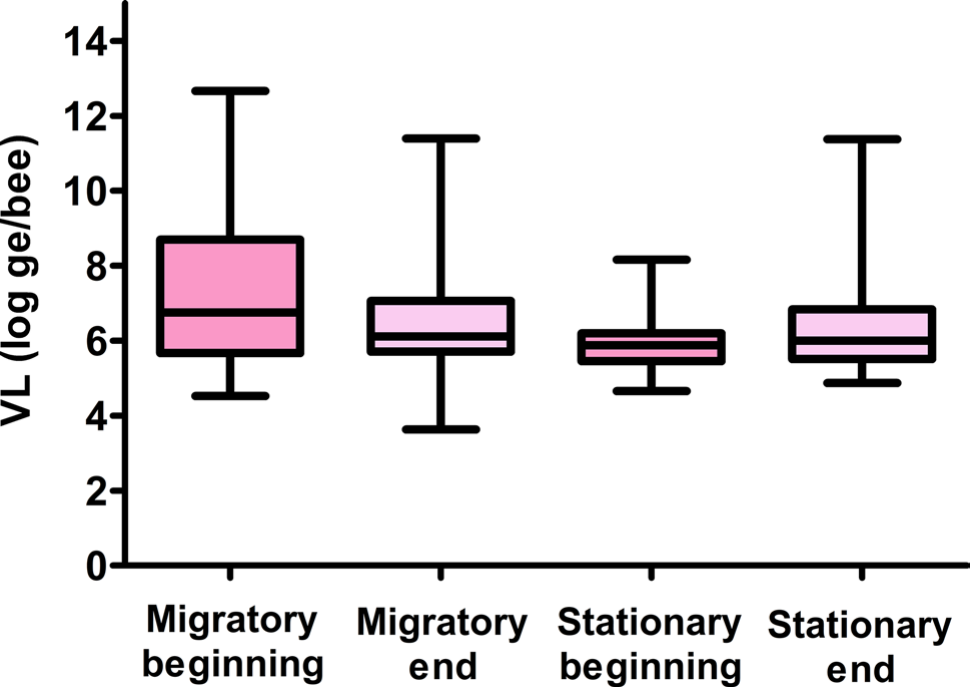
Quantification of DWV loads in migratory and stationary apiaries. DWV loads detected by RT-qPCR in migratory and stationary colonies during the beginning and end of the season. Viral loads (VLs) are expressed as Log10 DWV genome equivalents/bee.

Immediately after returning from native flowering, migratory colonies showed a decrease in the number of DWV-positive colonies. At that time, colonies under both types of management had similar DWV VLs (Table 2, Fig. 3).

**Table 2:** DWV detection in hives assayed by RT-qPCR, according to the type of management and sampling time.

Sample	Management	Season	Positive	%	Number of Hives	Mean VL (log_10_ genome equivalents/bee)
BEES	Stationary	Beginning	15	44.11	34	5.88
		End	17	53.12	32	6.70
	Migratory	Beginning	25	86.20	29	7.54
		End	33	66.00	50	6.42

The presence of DWV was also evaluated in larvae and pupae. A high percentage of DWV-positive samples was detected in *Varroa destructor* mites, across all types of management and sampling times. However, in larvae and pupae, a greater number of DWV-positive samples were found in migratory colonies compared to stationary ones, both at the beginning and end of the season (Table 3).

**Table 3:** DWV detection in pools from frames of larvae and pupae collected during the beginning and the end of season in apiaries with different types of management.

Sample	Management	Season	N° Positive	%	N° Pools
Parasitized larvae and pupae	Stationary	Beginning	2	28.57	7
		End	5	55.56	9
	Migratory	Beginning	25	80.64	31
		End	22	95.65	23
Non Parasitized larvae and pupae	Stationary	Beginning	5	62.50	8
		End	5	50.00	10
	Migratory	Beginning	18	78.26	23
		End	17	77.27	22
*Varroa destructor*	Stationary	Beginning	6	85.71	7
		End	7	77.78	9
	Migratory	Beginning	23	79.31	29
		End	19	86.36	22
Pollen	Stationary	Beginning	0	0.00	2
		End	2	28.57	7
		Wintering	2	50.00	4
	Migratory	Beginning	1	25.00	4
		End	0	0.00	5
		Wintering	8	80.00	10

Bee bread samples (pollen) were also tested for the presence of DWV and in 13 out of 32 samples the virus was detected. Notably, DWV was found in samples collected in July, suggesting that the virus remained and/or circulated in the colonies during wintering (Table 3 and Supplementary material 2).

### Association between DWV presence and monitored factors

The relationship between the type of management and various factors, including, DWV point prevalence, the presence of other pathogens, colony health, nutritional state, dietary supplementation, and time of sampling, was subjected to further analysis.

**Figure 4:**
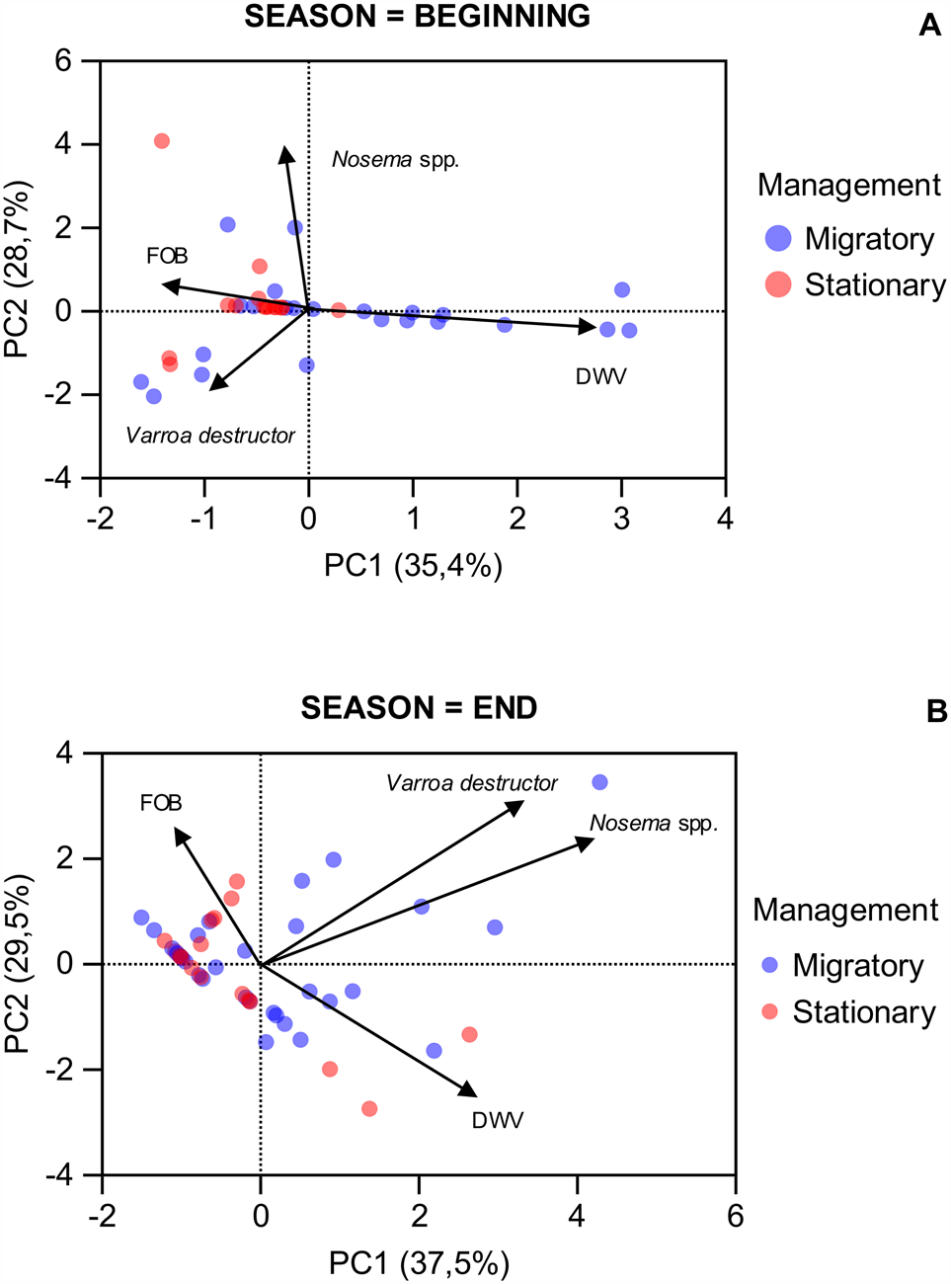
Principal component analysis. Biplot of the colonies showing dissimilar dispersion of stationary and migratory managements for the beginning (A) and the end (B) of the season, according to each variable: frames of adult bees, % foretic *V. destructor*, *Nosema* spp. spore counts and DWV loads. Stationary colonies are represented in rose, while migratory colonies are in blue. PC: principal component.

A PCA analysis, incorporating variables such as the number of frames of adult bees, *Varroa destructor* infestation rate, *Nosema* spp. infection level, and DWV viral load at the beginning and end of the season for both management types, enabled us to assess the relative weight of each variable in clustering the samples over the study period. The eigenvalues of the two principal components explained 64,1% and 67% of the overall variability, as shown in Fig. 4A,B, respectively. The DWV VLs at the beginning of the season in migratory management exhibited the strongest influence on colony dispersion in the PCA (Fig. 4A).

At the beginning of the season, the number of DWV-positive samples was significantly higher in migratory colonies than in stationary ones (p = 0.0005). Significant differences were also observed when hive category, Nosema spp. infection level and nutrition type were analyzed (p = 0.0002, p = 0.0252 and p = 0.0551 respectively). On the other hand, at the end of the season, Nosema spp. infection state, nutrition type and dietary supplementing conditions were the variables that showed significant differences between colonies subjected to stationary or migratory management (Table 4).

**Table 4:** Summary statistics of each variable at the beginning and the end of the season, for migratory versus stationary management.

Season	Variable	Level	Stationary apiary	Migratory apiary	χ^2^	p-value
Beginning	DWV detection	Positive	0.44	0.86	11.96	0.0005*
		ND	0.56	0.14		
	Hive Category	C1	0.91	0.62	17.16	0.0002*
		C2	0.09	0		
		C3	0	0.38		
	*Varroa destructor* Infestation	ND	0.71	0.79	3.98	0.2633
		Low	0.09	0		
		Medium	0.20	0.17		
		High	0	0.04		
	*Nosema* spp. Infection	ND	0.91	0.69	5.01	0.0252*
		Weak	0.09	0.31		
				0.62		
				0.35		
	Nutrition	Regular	0.47	0.03	5.80	0.0551*
		Good	0.35			
		Very Good	0.18			
	Dietary supplement	Yes	1	1	0.40	0.5287
		No	0	0		
End	DWV detection			0.66		
		Positive	0.53	0.34	1.36	0.2437
		ND	0.47			
	Hive Category	C1	0.66	0.5	3.14	0.2082
		C2	0.09	0.24		
		C3	0.25	0.26		
	*Varroa destructor* Infestation	ND	0.66	0.72	1.77	0.6221
		Low	0.06	0.1		
		Medium	0.22	0.16		
		High	0.06	0.02		
	*Nosema* spp. Infection	ND	0.88	1	4.14	0.0418*
		Weak	0.12	0		
	Nutrition	Regular	0.19	0	21.72	< 0.0001*
		Good	0	0.36		
		Very Good	0.81	0.64		
	Dietary supplement	Yes	0.34	1	44.11	< 0.0001*
		No	0.66	0		

Results of Pearson’s Chi-squared test for each variable among migratory and stationary management at the beginning and the end of the season. Significant differences were denoted by “*” at alpha < 0.05. Proportions for each level are shown on the different variables. ND: not detected.

Significance values are in bold.

Based on these findings, a generalized linear mixed effect model (GLMM) was conducted to analyze the impact of each variable (number of frames of adult bees, *Nosema* spp. infection level, and DWV VL) on the management at each sampling time. A positive association between DWV abundance and migratory management was found at the beginning of the season (p = 0.0613) (Table 5).

**Table 5:** Explanatory factors for migratory management at the beginning and the end of the season.

Season	Variable	Estimate (SE)	Z-value	p-value
Beginning	Intercept	− 5.12(0.98)	− 5.21	** < 0.0001**
	DWV loads	0.11(0.06)	1.87	**0.0613**
	FOB	− 0.15 (0.09)	− 1.58	0.1133
	*Nosema* spp. Infection	3.5E-06 (5.1E-06)	0.69	0.4902
End	Intercept	− 5.38(0.50)	− 10.78	** < 0.0001**
	DWV loads	0.01 (0.04)	0.33	0.7384
	FOB	− 0.02 (0.06)	− 0.42	0.6741
	*Nosema* spp. Infection	2.4E-06(2.8E-06)	0.86	0.3922

GLMM with binomial distribution for migratory management, random factor ‘apiary’. n = 145 colonies, SE: standard error. FOB: frames of adult bees.

Significance values are in bold.

Finally, an assessment of the risk associated with the variable that exhibited significant differences in the previous model, namely DWV VL in migratory management at the beginning of the season, was conducted. It was found that migratory management significantly increased (p = 0.031) the probability of having high DWV loads, with an increase factor of 1.98% (Odds Ratio = 1.98).

### Pollen identification

Pollen samples were useful in identifying the floral resources from which the colonies benefited, providing evidence of clear differences between the two types of management. In stationary apiaries, the most abundant species found in bee bread were native ones. At the beginning of the season, available resources came from native (such as *Schinus* sp.*, Celtis* sp.) or exotic (like *Gleditsia* sp., *Melia azedarach*) trees or shrubs, or adventitious (such as *Trifolium repens*, *Brassicaceae*) and cultivated (for example: *Vicia* sp.) herbaceous plants. By the end of the season, the pollen found belonged primarily to native species such as the *Trithrinax campestris* palm, shrubs (*Baccharis*), or herbs (*Senecio* sp.*, Grindelia*, *Bidens*) with a lesser extent contributions from cultivated forage such as alfalfa (*Medicago sativa*).

For migratory apiaries, at the beginning of the season, the most abundant resource came from *Citrus* spp., with a smaller contribution from native plants, both herbaceous (such as *Senecio* sp. and *Cyperus)*, and arboreal (for example: T. *Myrcianthes cisplatensis*). Due to the restricted variety of flowers in monoculture settings, beekeepers moved their apiaries to regions with richer floral diversity, offered by native plant species. However, by the end of the season, *Eucalyptus* spp. plantations became the primary floral resource for colonies in those areas, with additional contributions from native species like *Baccharis and Trithrinax campestris* (Supplementary material 3).

### Phylogenetic analysis

**Figure 5:**
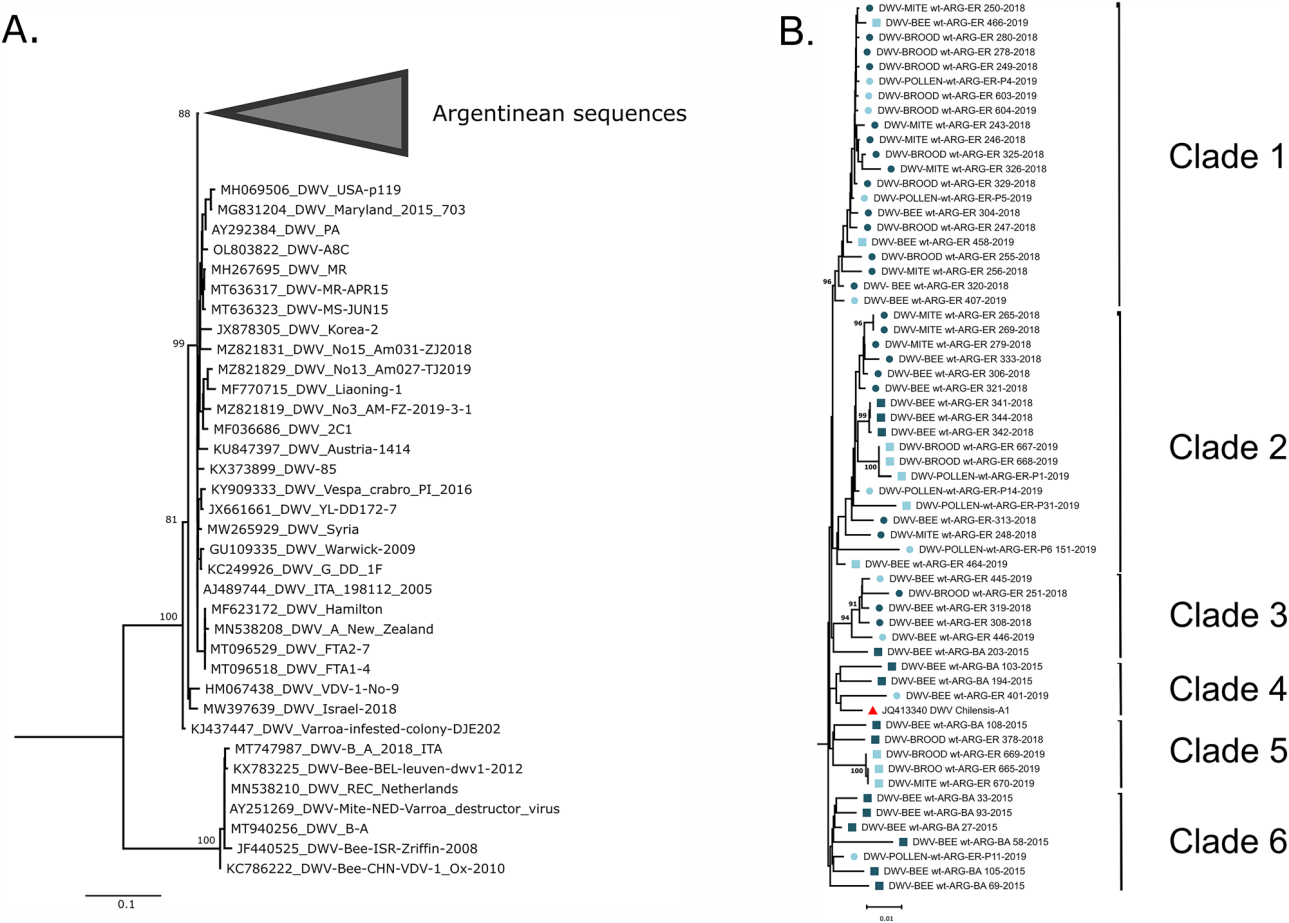
Phylogenetic analysis of DWV. A fragment of 1500 nucleotides (positions 5083–6583) of the DWV helicase gene was amplified and sequenced from 60 samples that belonged to migratory and stationary apiaries. (**A**) Neighbor-Joining phylogenetic tree showing the clustering of Argentinean sequences () related to reference sequences from different countries. (**B**) Phylogenetic tree of Argentinean DWV-A sequences from migratory colonies at the beginning (blue circles) and the end (light blue circles) of season, and from stationary colonies at the beginning (blue squares) and the end (light blue squares) of the season. The red triangle refers to a whole genome sequence from Chile.

To identify the type of DWV variants circulating in the study areas, a phylogenetic analysis was conducted which revealed that all Argentinean sequences belonged to DWV type A (helicase) and clustered together in a monophyletic and highly supported branch (bootstrap = 100) (Fig. 5A).

Sequences from Villa del Rosario (migratory management) were mainly included in three well-supported clades (clades 1, 2, and 3). Clade 1 primarily consisted of samples from migratory apiaries, including those collected at the beginning and end of the season, derived from adult bees, larvae and pupae, or *V. destructor* mites. Particularly, a distinct branch emerged, uniting samples isolated from stationary hives at the end of the season, which had interacted with migratory colonies. Clade 2 included sequences from both migratory and stationary apiaries, irrespective of the sampling time. This lack of clustering could be attributed to the sharing of feeding sites during a specific period. Clade 3 consisted of sequence BA-203–2015 from a stationary apiary located in Buenos Aires, and sequences from migratory apiaries collected at the beginning and the end of the season. Notably, these migratory apiaries ventured into the border region between the provinces of Buenos Aires and Entre Ríos. Three additional clades were also evident from the analysis. Clade 4 grouped samples isolated in 2015 from stationary apiaries located in Buenos Aires, along with a migratory one from Entre Ríos, which was related to the sequence DWV-Chilensis-A1, from Chile. Clade 5 included exclusively samples from stationary apiaries collected in Entre Ríos at the beginning and the end of the season. Clade 6 was primarily composed of samples from stationary apiaries from Buenos Aires, except for a pollen-isolated sequence, ER-P11-2019 (Fig. 5B).

## Discussion

Argentine is renowned for the production of clear honeys benefiting from diverse landscapes across its territory. However, the intensification of agriculture has brought about the existence of multifloral or monofloral areas, leading to changes in beekeeping production scenarios. Consequently, beekeepers employ either migratory or stationary management strategies to enhance honey production, depending on flowering patterns.

Worldwide, migratory beekeeping is practiced for either performing pollination services or enhancing honey production. Despite the benefits provided by this practice, recent studies have highlighted the potential drawbacks of migratory beekeeping. In particular, this activity increases the risk of acquiring and spreading pathogens and parasites, which can have detrimental effects on colony health^19^. The presented results on detected DWV infection may act in support of this concern.

Regarding the detection of *Nosema* spp., no significant differences were observed between the type of management or the time of sampling. Concerning the *Varroa destructor*, low percentages of infestation in the phoretic stage were detected at any time of sampling (beginning vs. end of the season) and irrespective of the type of management (migratory vs. stationary). It is well reported that migratory management negatively impacts on mite infestation^18,20^, which motivates the rigorous control of *Varroa destructor*. The low level of *V. destructor* found in our study can be attributed to the strict control management applied to the colonies. It is worth noting that samples for *Varroa destructor* detection were collected before the routine treatment against the mite was applied.

When examining the brood, *V. destructor* mites were detected in both migratory and stationary apiaries, with a higher number of mites found in the brood from migratory apiaries. This indicates that mites were present in both areas, even in colonies with non-detectable phoretic mites, and suggests that a significant portion of the mites present at the time of sampling were in the reproductive stage^41,42^. However, it´s important to note that our study cannot definitively determine whether the type of management influences the presence or spread of these pathogens, as observed in other studies^18,19^. On the other hand, stationary apiaries in the study region have adapted to the presence of migratory colonies in neighboring areas and have increased control measures against these pathogens to prevent infections and infestations from non-local apiaries. Therefore, levels of *Nosema* spp. and *V. destructor* were low and similar in both types of management.

The presence of DWV was detected in 62.07% (90/145) of the processed samples. This high percentage of positive samples is consistent with other reports in various regions of the country^43,44^. We found statistically significant associations between the presence of the virus, the sampling time, and the type of management, particularly in migratory apiaries at the beginning of the season (p = 0.0005). This result may be a consequence of the negative impact experienced by apiaries located in areas dominated by monoculture. Adequate nutrition is known to improve and maintain colony health, and it´s worth noting that the nutritional content of pollen varies by geographic region^16^. Therefore, pollinators in monofloral crop areas, with reduced floral diversity and nutritional resources, are more susceptible to diseases^16,39,45^. In our study, honey bees from migratory apiaries primarily forage on *Citrus* spp. monocultures at the beginning of the season and *Eucalyptus* spp. at the end. Pollen samples collected at each sampling time from inside the colony confirm the dominance of these monocultures in their diet. This lack of floral diversity could potentially impact colony strength and, as a result, contribute to the increased circulation of DWV.

Recent studies conducted in *Eucalyptus grandis* plantations in Uruguay reported the negative impact of nutritional stress on colony strength. Beehives exposed to monoculture exhibited reduced population sizes, fewer broods, and increased disease susceptibility compared to colonies supplemented with multifloral pollen in their diet^46^. In our study, migratory hives returned to their area of origin (Villa del Rosario) for the flowering of *Eucalyptus* spp. at the end of the season, after a period of transhumance in multifloral areas. At that time, colonies presented 20% fewer DWV-infected colonies and lower VLs than at the beginning of the season. Additionally, the colonies showed increased strength, with only 24% of the colonies categorized as weak (C3), 26% as average (C2), and 50% as strong (C1). These results highlight the benefits of the nutritional diversity offered by the flowering of native forests. Subsequently, colonies stayed in Villa del Rosario during the flowering period of *Eucalyptus* spp. until wintering. While this enables beekeepers to harvest a specific variety of honey, colonies enter the winter season with the stores of monofloral pollen from *Eucalyptus* spp. This species has low lipid content, a low percentage of crude protein, and is deficient in isoleucine^46–48^, consequently, failing to meet the minimum requirements for colony maintenance and breeding. As a result, beekeepers must add a dietary supplement to ensure that the colony has sufficient reserves to survive the winter.

It is worth noting that sedentary and migratory colonies were subjected to different honey extraction schedules, with an extra harvesting in migratory colonies after the flowering period of *Eucalyptus* spp., which could impact the colonies´ nutrition supplies.

This suggests that the advantages of migration, aimed at enhancing floral diversity and improving colony health may be nullified by staying in a monofloral area before wintering, which becomes evident at the beginning of the following spring. In support of this assumption, at the beginning of the season, migratory colonies in our study exhibited a high proportion of DWV-positive samples (86.2%) and elevated viral loads in adult bees. Furthermore, 38% of these hives were categorized as C3, indicating a weakened condition in terms of their population size.

We considered the lack of nutritional diversity as a potential factor affecting colony health in our study, as it has been described in other reports^49,50^. However, this assumption must be considered cautiously, and further analysis of the protein contribution provided by the pollen is required.

For the stationary apiaries located in the central region of the Entre Ríos province, the high floral diversity enables varied pollen intake into the colonies^51^, promoting colony health at the beginning of the season with a majority classified as C1 (strong health condition, 91%). However, by the end of the season, after a period of coexistence with migratory hives, there was a reduction in strong colonies (66% classified as C1), an increase in weak ones (25% classified as C3), and a higher number of DWV-positive colonies. These results may be a consequence of the migratory movement, as bees from both colony types share floral resources and feeding sites, potentially enhancing the horizontal transmission of pathogens^52^.

While the presence of viruses affecting honey bees has been reported in various provinces of Argentina^43^, the identification of circulating DWV A and B variants was recently reported in apiaries located in Buenos Aires and Santa Fe provinces. Given the limited information available regarding DWV in our study area, we aimed to determine the DWV variant present in the collected samples. The sequence corresponding to the helicase of DWV A master variant was identified in 60 samples obtained from adult bees, parasitized larvae and pupae, non-parasitized larvae and pupae, pollen and *V. destructor* mites. Furthermore, we sequenced the entire genome of two samples, through next generation sequencing and confirmed that the master variant present was DWV A (GenBank: OR597290.1)^53^. All the Argentine sequences exhibited a common geographic structure, as they did not cluster with any other sequence worldwide, except for one from Chile.

Interestingly, the sequences obtained from both symptomatic and asymptomatic samples exhibited a 99% amino acid similarity, indicating the presence of the same circulating variant. This suggests that the appearance of desease signs in symptomatic bees was caused by the same virus variant detected in asymptomatic samples.

In conclusion, while migratory management in Argentina is designed to enhance honey production and achieve diverse honey varieties, it necessitates careful consideration, especially when colonies conclude the season in monofloral areas. Such colonies may enter winter in suboptimal conditions, resulting in weakened states with an increasing risk of an elevated DWV prevalence and abundance at the onset of the following season. Therefore, it is imperative for beekeepers to implement migratory practices judiciously to ensure the health and vitality of their colonies.

## Methods

### Sampling strategy

During the period from September 2018 (early spring in the southern hemisphere) to March 2019 (early autumn in the southern hemisphere), two samplings were carried out in the Entre Ríos province, Argentina. The apiaries were located in two different zones based on their management practices. Macia village is characterized by stationary operations (stationary apiaries), meaning that the colonies remain in the same location throughout the season (from September to March). In Villa del Rosario, migratory operations (migratory apiaries) are performed from December to February.

Beekeepers manage more than 1000 hives, with each apiary containing between 60 and 100 hives. All the apiaries were dedicated to honey production. In the migratory apiaries, honey was harvested at the end of each flowering season (October, January, March). However, for the stationary apiaries, the harvest took place between the end of spring and the beginning of autumn (October, March). The transport of honey bees was carried out following the requirements of the Argentine Animal Health Authority, which establishes that the colonies must not present any clinical signs associated with *American foulbrood* disease and infestation with small hive beetle or *Tropilaelaps* mite*.*

Beekeepers rigorously applied the synthetic acaricide Amitraz or Oxalic acid protocols for *Varroa destructor* control. In the stationary apiaries two treatments were conducted at the beginning and end of the season, while in the migratory apiaries treatments were applied at each migratory operation. In addition, queen replacements were conducted annually in all hives, typically in October.

### Sample collection

Sampling was conducted at two distinct time points: at the outset of the season in September 2018 and again at its conclusion in March 2019. These samplings occurred prior to the application of the acaricide treatment. A total of 145 hives, distributed across 15 different apiaries within stationary and migratory zones (66 hives from stationary apiaries and 79 hives from migratory apiaries), were included in the study (as detailed in Table 1).

Within each apiary, a minimum of six colonies were randomly selected for sampling. In apiaries with more than 60 hives, 10% of the total number of colonies were chosen for sampling. All collected samples were promptly shipped to the laboratory on dry ice and subsequently stored at -80°C until further analysis.

Approximately, 300 bees were collected from the frame of brood to detect and quantify virus and to calculate the percentage of phoretic mite infestation. The samples consisted of female bees representing various age groups, encompassing nurse bees, worker bees, and forager bees. For the detection of *Nosema* ssp., foraging bees were specifically collected at the hive entrance.

During each sampling event, a minimum of one hive per apiary was chosen for larvae and pupae analysis. A portion of the brood was carefully extracted to assess the presence of *V. destructor* and DWV. Additionally, pollen samples were collected. The presence of DWV was subsequently examined in larvae, pupae, mite, and pollen samples.

### Quantification of beehive health

Beehive health was evaluated using the classification described by Cayley Faurot-Daniels et al.^54^. Briefly, beehives were categorized as either strong (C1: category 1), average (C2: category 2), or weak (C3: category 3), primarily based on their population size. This assessment was determined by the number of frames occupied by bees. To account for variations in flower availability between the beginning and end of the season, the criteria for each category were adjusted according to the specific sampling time. At the beginning of the season in September 2018, hive categories were established as follows: weak (C3) represented hives with fewer than 5 frames covered by bees, average (C2) included hives with 5 to 7 frames covered by bees, and strong (C1) encompassed hives with 8 to 10 frames covered by bees. By the end of the season in March 2019, the criteria for each category were adjusted to reflect the number of frames covered by bees as follows: weak: fewer than 5 frames, average: 6 frames, and strong: 7 to 8 frames.

Furthermore, a visual inspection of the adult bee population and brood was conducted. During this inspection, the presence of disease symptoms such as bees with deformed wings, adult and larvae mortality, and significant reductions in the adult population were recorded. Additionally, the presence of *V. destructor* and the mortality rate per colony were assessed.

Similarly, hives were categorized based on their nutritional status, determined by the availability of food within the brood chamber. The classifications included “very good” (VG), denoting hives with four frames containing pollen and honey; “good” (G), signifying hives with two frames; and “regular” (R), indicating hives with only one frame of stored food. At the beginning of the season, all hives located in stationary and migratory zones received an identical dietary supplement, which consisted of pollen granules and sucrose syrup.

By the end of the season, all the hives were maintained at a level of 2 to 4 frames containing pollen and honey. This was achieved by providing additional dietary supplements to ensure the hives´ survival through the winter.

### Quantification of *Varroa destructor*

*Varroa destructor* infestation was monitored in each colony by analyzing 300 nurse bees collected from the frame of brood. This analysis followed the methodology outlined in Dietemann et al. (2013)^55^. The infestation level for each apiary was expressed as the percentage of mites found in the total sample analyzed (number of mites/bee * 100).

### Detection of *Nosema* spp. spores

The level of *Nosema* spp. infection within each colony was determined by examining a pool of 60 bees collected at the hive entrance. The spore count was conducted according to the method described by Catwell^56^ and modified by Fries^57^. Level of infection was expressed as number of spores per bee.

### Detection and quantification of DWV

For RNA extraction, a group of 30 bees collected from the central brood frames was processed. To analyze parasitized and non-parasitized larvae and pupae, pooled samples of 10 individuals from each respective category were examined. *V. destructor* samples obtained from the parasitized larvae and pupae were analyzed based on the level of infestation: if the brood presented fewer than 5 mites, they were combined into a single pool; otherwise, when more than 5 mites were found per brood, pools consisting of 5–7 mites were created.

All bee, larvae and pupae samples were processed by macerating them in a mortar with an appropriate volume of PBS pH 7 (7 ml for adult bees and 3 ml for larvae and pupae). The *V. destructor* samples were first frozen with liquid nitrogen and subsequently macerated in a mortar with 0.5 ml of PBS at pH 7. Bee bread was collected in those frames where larvae and pupae samples were taken. The material was extracted from 50 cells, resulting in one pool per hive. Each pool consisted of 100 mg of material, which was resuspended in 0.5 ml of PBS at pH 7.

The complete mixtures were centrifuged at 2717× g at 4 °C for 45 min. From the resulting supernatant, 200 µl were utilized for RNA extraction using the High Purity Viral RNA Kit (Roche) and following the manufacturer's recommendations. The remaining supernatant was collected and stored at – 80 °C.

cDNA synthesis was performed using M-MLV Reverse transcriptase (Promega). The reaction mixture contained 5 µl of RNA, 5 µl of reaction buffer 5× (Promega), 0.5 µl of dNTP 10 mM (Promega), 0.5 µl of random primers 2 µg/µl, 0.25 µl of reverse transcriptase 200 U/µl, and 13.75 µl of ultra-pure water (Distilled Water DNAse, RNAse Free; Invitrogen/Sigma) to obtain a total volume of 25 µl. Reverse transcription was performed at 42 °C for 45 min followed by a denaturation step at 94 °C for 10 min to inactivate the reverse transcriptase.

The viral load of Deformed Wing Virus (DWV VL) was assessed by quantitative PCR using primers that were previously descripted by Bradford et al. 2017 (Pan-DWV)^29^. This set of primers was designed to amplify a region of 179 bp within the helicase protein, enabling the detection of all DWV variants.

All qPCRs were performed in a final volume of 12.5 µl containing 6.25 µl iTaq Universal SYBR Green Supermix (Bio-Rad), 0.5 µl of each primer (10 µM), 2.75 µl H_2_O and 2.5 µl of cDNA template. The thermal qPCR profile included an initial denaturation step of 3 min at 95 °C, followed by 40 cycles of amplification, each consisting of 15 s 95 °C and 1 min of 60 °C. A melting curve analysis was performed, ranging from 65 to 95 °C, with the temperature increasing 0.5 °C every 5 s. A five-points standard curve was prepared with Ct data derived from known concentrations of a plasmid containing the target sequence. These concentrations encompassed tenfold dilutions, ranging from 7.3 × 10^6^ to 10^2^ genetic copies/µl). Each point on the standard curve was assayed in triplicate. Viral loads (VLs) were experessed as log10 genome equivalents/bee. The housekeeping gene β-actin was amplified as an internal control using the primers described by Chen et al.^58^.

### Statistical analysis

Initially two descriptive methodologies were carried out, a principal component analysis (PCA) to compare the relative weight of the different quantitative variables and a chi-square test to compare categorical variables such as DWV point prevalence, *Nosema* spp. and *Varroa destructor* presence, nutrition, dietary supplement, and hive category (according to population size).

Variables that exhibited significant differences between migratory and stationary colonies were then included in the construction of a generalized linear mixed effect model (GLMM). This model was employed to investigate the impact of each variable, including the number of frames of adult bees (FOB), *Nosema* spp. infection level, and DWV viral load. Models were compared by ANOVA’s, and the Akaike Information Criterion (AIC) was considered as an additional criterion for selection.

Finally, to evaluate the risk of the variable presenting significant differences in the previous model, another generalized linear mixed effects model (GLMM) was carried out. The analysis was conducted considering migratory management at each time of sampling (ie beginning and end of the season).

All statistical analyses were performed using InfoStat software (Version 2020). Figures 2 and 3 were performed using Graphpad Prism 10.1software.

### Pollen identification

The examination of pollen collected from both migratory and stationary apiaries was conducted through the analysis of bee bread. To process the bee bread, the protocol described by Fagúndez et al.^59^, was followed. For observation and analysis under an optical microscope, the pollen residue was subjected to acetolization, following the methodology originally described by Erdtman et al.^60^. The contribution of each type of pollen in the diet of bees was calculated using the method outlined by O'Rourke and Buchmann et al.^61^.

### Sequencing

Samples testing positive for DWV were selected for sequencing. These samples included bees, larvae, pupae, *V. destructor*, and pollen. Additionally, DWV-positive samples from stationary apiaries located in the province of Buenos Aires, which had been previously analyzed in our laboratory, were also included in the study. The selected samples were amplified by RT-nPCR using the primers described by Ryabov et al.^30,62^ that targeted a 1600 bp fragment within the helicase coding region. Briefly, cDNA synthesis was performed as previously mentioned. All nested PCRs were carried out using the Gotaq polymerase (Promega). The reaction mix contained 5 µl cDNA, 5 µl 5× reaction buffer (Promega), 1.5 mM magnesium chloride, 0.5 µl 10 mM dNTPs (Promega), 0.5 µl primers (10 µM), 0.25 µl 5U/µl polymerase, and 13.75 µl ultrapure water (DNase distilled water, RNase-free; Invitrogen/Sigma), in a total volume of 25 µl. Positive and negative controls were incorporated in all assays. The thermal nPCR profile was as follows: an initial denaturation step of 5 min at 95 °C, followed by 35 cycles, each consisting of 30 s at 95 °C, 45 s at 52 °C, and 1 min at 72 °C, concluding with a final elongation step of 7 min. Purification of the PCR products and sequencing by the dideoxynucleotide chain termination method were performed using the services of Macrogen Inc (City, Korea).

Sequences generated in this study were deposited into GenBank (Supplementary material S4).

### Phylogenetic analysis

Datasets were constructed with the most related strains identified through Basic Local Alignment Search Tool (BLAST) analysis on the NCBI website (https://blast.ncbi.nlm.nih.gov/). A total of 95 strain sequences were compilated to create the dataset for DWV. Subsequently, multiple sequence alignments based on 1500 nucleotides (spanning positions 5083 to 6583) were performed and edited using Muscle available in AliView v1.16 (Larsson)^63^. Before proceeding with the phylogenetic analysis, a quality assessment of the dataset was performed. Briefly, the phylogenetic information was estimated using IQ-Tree^64^ and the PHI test for recombination was carried out in Split-Tree4^65^. Phylogenetic and molecular evolutionary analyses were conducted using MEGA version X (Kumar et al.^66^). The Neighbor-Joining (NJ) method (Saitou and Nei^67^) was used. The evolutionary model selected based on the Bayesian Information Criterion (BIC) was Tamura-Nei (TN93); this model included a discrete Gamma distribution (+G) with 5 rate categories, and it assumed that a certain fraction of sites is evolutionarily invariable (+I), which is referred to as the TN93 + G + I. The branch supports were estimated using the bootstrap method including 1000 pseudo-replicates. All positions with less than 95% site coverage were eliminated, meaning that fewer than 5% alignment gaps, missing data, and ambiguous bases were allowed at any position (partial deletion option = 95%).

### Supplementary Information

Supplementary Information.

## Data Availability

Sequences generated in this study were deposited into GenBank and are specified in Supplementary material S4.
